# A Multi-Agent Formalism Based on Contextual Defeasible Logic for Healthcare Systems

**DOI:** 10.3389/fpubh.2022.849185

**Published:** 2022-03-03

**Authors:** Salwa Muhammad Akhtar, Makia Nazir, Kiran Saleem, Rana Zeeshan Ahmad, Abdul Rehman Javed, Shahab S. Band, Amir Mosavi

**Affiliations:** ^1^Computer Science Department, Faculty of Computer Science and IT, University of Lahore, Lahore, Pakistan; ^2^School of Software, Dalian University of Technology, Dalian, China; ^3^Department of Information Technology, University of Sialkot, Sialkot, Pakistan; ^4^Department of Cyber Security, Air University, Islamabad, Pakistan; ^5^Future Technology Research Center, College of Future, National Yunlin University of Science and Technology, Douliou, Taiwan; ^6^Institute of Information Society, University of Public Service, Budapest, Hungary; ^7^John von Neumann Faculty of Informatics, Obuda University, Budapest, Hungary; ^8^Institute of Information Engineering, Automation and Mathematics, Slovak University of Technology in Bratislava, Bratislava, Slovakia

**Keywords:** healthcare system, NetLogo, web ontology, multi-agent system, medical internet of things (IoT)

## Abstract

In the last decade, smart computing has garnered much attention, particularly in ubiquitous environments, thus increasing the ease of everyday human life. Users can dynamically interact with the systems using different modalities in a smart computing environment. The literature discussed multiple mechanisms to enhance the modalities for communication using different knowledge sources. Among others, Multi-context System (MCS) has been proven quite significant to interlink various context domains dynamically to a distributed environment. MCS is a collection of different contexts (independent knowledge sources), and every context contains its own set of defined rules and facts and inference systems. These contexts are interlinked *via* bridge rules. However, the interaction among knowledge sources could have the consequences such as bringing out inconsistent results. These issues may report situations such as the system being unable to reach a conclusion or communication in different contexts becoming asynchronous. There is a need for a suitable framework to resolve inconsistencies. In this article, we provide a framework based on contextual defeasible reasoning and a formalism of multi-agent environment is to handle the issue of inconsistent information in MCS. Additionally, in this work, a prototypal simulation is designed using a simulation tool called NetLogo, and a formalism about a Parkinson's disease patient's case study is also developed. Both of these show the validity of the framework.

## 1. Introduction

Ubiquitous computing deals with invisibly interweaving the real world with various agents embedded seamlessly in everyday objects of lives and connected through dedicated networks and media to make our everyday life easier and more efficient (1–3). When the agents were initially designed, they were used solely to detect user location, however, with technological advancements, they gradually were able to adapt to interfaces, increase the precision of information retrieval, discover services users may require, and most importantly build smart environments (4). Smart environments are the physical environments used in daily human life that are seamlessly embedded with tiny smart devices equipped with sensors, actuators, and computational elements (agents). These physically embedded devices are connected through a continuous data collection and processing network to enable various pervasive applications, services, and smart environments to perform efficiently. The usage of various connected agents for data retrieval, processing, and exchanging make up a system called Multi-Agent System (MAS) (5).

A multi-agent system is a computerized system comprising multiple interacting intelligent agents (6). These intelligent agents can collect data, process it, and then send it to the controller/central system for further analysis. While this level of intelligence and functionality is more than enough for some applications, for others, this is not. These are the applications or systems that needed information from multiple resources (domains) to execute their required operations (4). This problem leads to a solution termed Multi-Context Systems (MCS).

Equilibrium is defined as a belief state that comprises a belief set, consisting of beliefs of each context in the MCS, such that an MCS is said to be in equilibrium if all the heterogeneous contexts have consistent beliefs (7). Inconsistency is a significant concern depriving MCS of reaching its full potential. Inconsistency makes it impossible for the system to conclude from the provided data. Suppose a system cannot derive a decision, then it is considered a failure. This work tries to ensure that the system maintains its equilibria state. This is the state in which each element of the system has a single belief, i.e., the decision should be the same according to each element of the system. The equilibria are lost whenever the system receives contradictory or inconsistent information. This makes the overall system not provide a conclusion, causing it to become purposeless. This becomes a severe issue, especially in the safety-critical system dealing with life and death situations in healthcare systems (8).

The proposed system uses a case study about a Parkinsons' disease patient to show its correctness. The system includes a Multi-Context system (MCS) consisting of several heterogeneous contexts. Several agents acquire information, process data, and perform reasoning accordingly in each context in MCS. As physical implementation of such a system is not feasible due to financial and technological constraints, virtual agents model the proposed smart environment. It is assumed that these virtual agents are intelligent; thus, they are intelligent virtual agents. Virtual agents can also be considered a type of software that collects, processes, and shares data just like a physical sensor and agent. It is assumed that data is acquired from virtual sensors for data acquisition. In the proposed framework, intelligent agents acquire contextual information from the sensors and perform reasoning accordingly. For each domain, an ontology is developed using Protégé, a free, open-source ontology editor and management system. Each of these ontologies is connected using mapping rules. These ontologies are mapped on their corresponding description logic. These description logic-based ontologies are then linked together using distributed description logic (DDL) formalism. Once the system gets settled, the flow of information begins due to the communication between the heterogeneous contexts. After this, the system's conflicting information is dealt with using CDL (9). Contextual defeasible reasoning can handle inconsistencies occurring due to contradictory information (8).

The rest of this article is structured as follows. Section Related Work presents the related work. Section Contextual Defeasible Reasoning (CDL) Based Multi-Agent Formalism presents the extensive description of the architecture of the framework proposed and the inclusion of Contextual Defeasible Reasoning in it. Section Framework Development discusses the contexts developed for the framework and their corresponding ontologies. Section Temporal Logic Formalism covers the formalism of the proposed work using temporal logic. Section Simulation of Proposed System discusses the simulation of the system done using NetLogo. In section Algorithm the proposed algorithm for the system is discussed in detail. Finally, this work is discussed overall and concluded in sections Limitations and Future Scope and Conclusion, respectively.

## 2. Related Work

Authors in (9) proposed a methodology for handling inconsistency produced in a Multi-Context System (MCS) by utilizing the consistency-based and abduction-based techniques. In the first approach, pairs of sets of mapping rules are considered, such that deactivating the rules in one set and forcing the rules in the other to be active allows to re-establish consistency. While in the second approach, a pair of rules were considered whose joint (de-)activation reproduced the observed inconsistency. In (8), the authors proposed a framework based on Contextual Defeasible Logic (CDL) for distributed MCS. The authors deployed the Meta-rules technique to perform reasoning on the system-acquired data.

In (10), the authors present a smart city ontology called KM4City (Knowledge Model for City). This ontology includes data coming from various heterogeneous sources and mapped onto it. Since ontologies are flexible, therefore, the addition of new information can easily be managed. Static and dynamic data from various heterogeneous sources is obtained and stored in RDF syntax, accessed using SPARQL queries. This data was from public and private sources, such as people sending information from their devices. The primary purpose of the author's KM4City is to help citizens on roads, mainly those stuck in traffic, and especially helping the ambulances find an optimal path to their destinations, be it the hospital or the location of a patient. The proposed work, according to the authors, overcomes the gap of combining public and private data from various heterogeneous sources. Various applications like those of public administrations and enterprises can be made using this framework. The information collected for the scope of the proposed paper was limited to road graphs, services available on the roads, and traffic sensors.

In (11), the authors have used an RPI-CAM-V2 pi camera, soil moisture sensor, and a temperature sensor and connected them to a Raspberry pi microcontroller. A raspberry pi is a circuit board that can be attached to a computer monitor through the USB port. This system has especially been developed for the farmers or the caretakers of agricultural lands to increase the efficiency of the agricultural systems by automating the irrigation process. It then sends the data to the Thingspeak mobile application (an android application available on the play store of all android devices), where the farmers can easily have access to the environmental conditions of the fields without being in the area. The authors have also used the Telegram mobile application for fetching data from raspberry pi when in remote locations. Telegram application is a messaging application similar to Whatsapp, except for creating bots that interact with both humans and machines.

In (12), the authors have presented a survey on the application of technologies of industry 5.0 in different domains. Industry 5.0 is mainly used to increase the efficiency and effectiveness of large-scale product manufacturing. However, like any other technology, this also comes with many drawbacks, mainly privacy and security dilemmas. In (13), the authors have presented a grid and place neuron model in cognitive tasks applications. The 2D virtual environment was created to deploy this system, which resulted in 92.27 % localization accuracy.

In (14), the authors have highlighted the usage of ML algorithms for the diagnosis, analysis, and prediction of stroke. Moreover, the authors used the Antlion optimization (ALO) algorithm using a deep learning model in minimal time consumption for optimal hyper-parameters selection. The training time for the proposed model is 38 % as a positive outcome considered for the experimental superiority results.

In (15), the authors have proposed a novel pairing-free certificateless scheme using blockchain technique and a CLS scheme using a smart contract. After this, they simulated the Type-I and Type-II adversaries to verify their scheme. After a thorough analysis, the authors deduced that their work reduced 40.0% computation cost and 94.7% communication cost. In (16), the authors have highlighted the emergence of heterogeneous Internet of Medical Things (IoMT) (e.g., for smart healthcare systems) that is used for sending huge bulk of patient's data for disease analysis to central cloud servers. However, it is prone to many security issues that can be overcome using AKA p. The authors have proposed an authentication protocol using blockchain technology and physically unclonable functions (PUF) in their work. In addition, biometric information is dealt with using a fuzzy extractor scheme. Their analysis proved that their work requires the most negligible computation and communication cost among the compared schemes.

This work is novel in the way that it proposes a smart decision-making healthcare system based on formalism technique that can help the medical professionals in their everyday routine and the patients living in far-off areas. The modeling of the system was done using ontologies so a clear picture of the system and the flow of information in it can be obtained. In order to check the validity of this work, a simulation was created using the NetLogo simulation tool. The existing approaches are summarized in Table 1. This work is different from others such that it uses contextual defeasible logic to resolve inconsistencies in multi-context systems. This technique is helpful when the entire system needs to be controlled automatically with the help of rule-based formalism in order to allow the system to reach a conclusion about the patient's health and provide the medical authority about the current situation.

**Table 1:** Analysis of related work.

**Reference**	**Technique**	**Domain**
(8)	Defeasible logic theory using the notion of meta-rules	Multi-context distributed systems
(10)	Ontology-driven modeling	Smart city
(14)	Machine learning algorithms	Smart healthcare
(9)	Consistency-based and abduction-based techniques	Multi-Context System (MCS)
(12)	Industry 5.0 supporting technologies	Internet of Everything (IoE)
(13)	Grid and place neuron model	2D virtual environment
(11)	Raspberry-pi circuit board	Smart agriculture
(16)	Internet of Medical Things (IoMT)	Smart healthcare
(15)	Blockchain technique	Internet of Things (IoT)
(17)	Elliptic Curve Digital Signature Algorithm (ECDSA)	Large-scale batch verification

## 3. Contextual Defeasible Reasoning (CDL) Based Multi-Agent Formalism

This section covers the CDL based on multi-agent formalism. In the context-aware MAS, every agent has a set of defeasible rules in their knowledge base where the contextual information and reasoning strategy is stored. Multi-context system notions are used for modeling heterogeneous systems (18–20). Multiple ontologies can send contextual information (e.g., rules, and facts). Each knowledge source sends the contextual information to its corresponding agents. After that, agents perform their reasoning mechanism based on the pre-defined rules. Reasoning types are two called, locally and globally. In local reasoning, the rules are performed by obtaining, through a single ontology. In distributed reasoning, multiple ontologies extract the sets of rules and facts by which each agent performs its reasoning. A framework of contextual defeasible reasoning based on heterogeneous formalism is proposed in this thesis. The framework uses the data acquired by sensors and analyzed and shared by agents, working in various heterogeneous contexts/domains with their respective systems. The following case study has been developed, which will form the basis of framework modeling and development.

**Figure 1:**
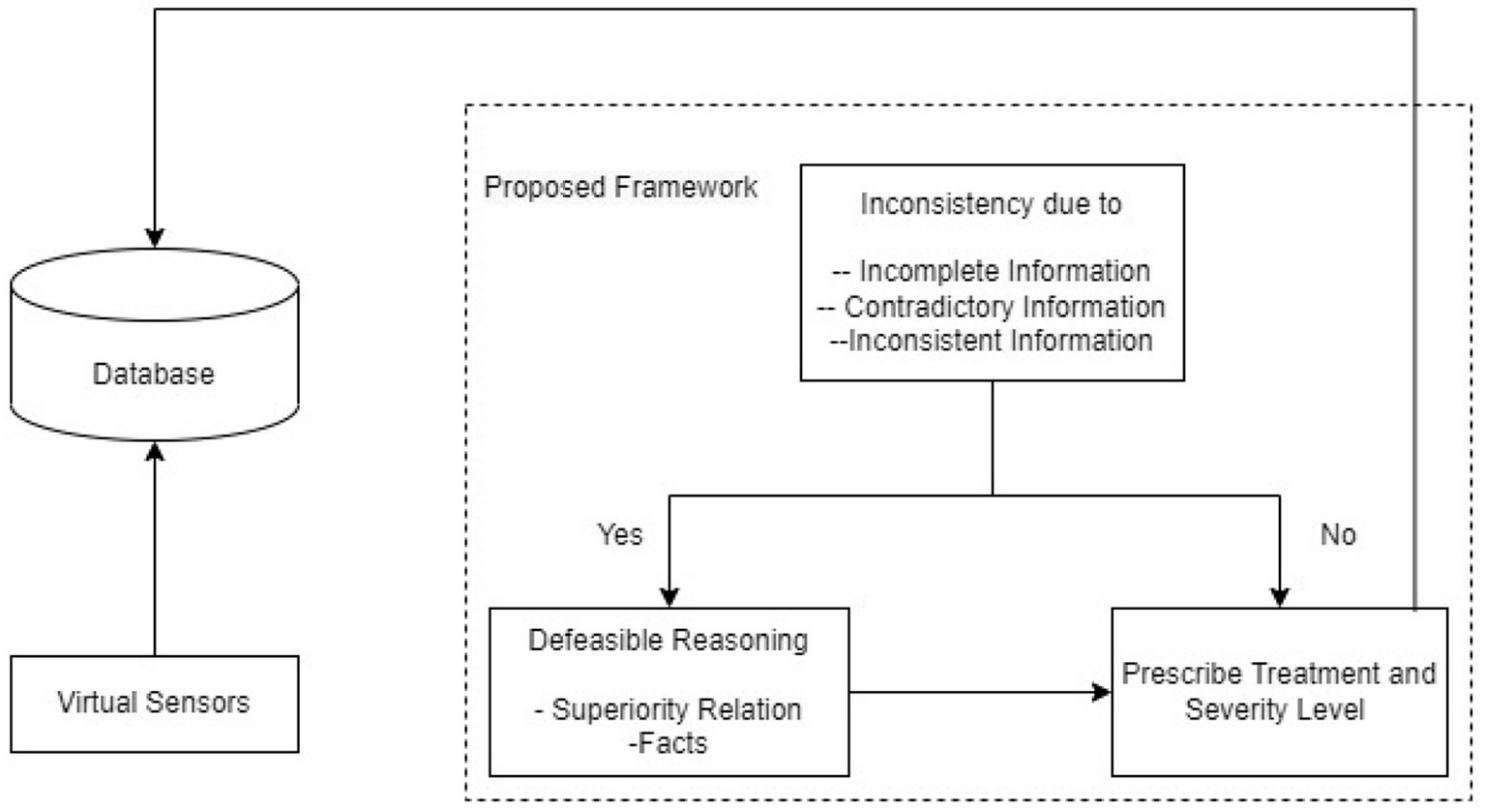
Architecture diagram.

Figure 1 shows the working of the proposed system. First, the virtual sensors continuously send data to a database. This database contains additional information about the patient, his allergies, and insurance. The information from the database (which also contains data from the sensors) is sent to the proposed system, which is the intelligent system responsible for making a decision. The intelligent system responsible for making a decision acquires this information and tries to derive a conclusion. In case of no inconsistency during conclusion making, then the system prescribes the treatment for the disease and keeps a record of the severity level of the patient. But if an inconsistency does occur, then the system first handles this inconsistency by using contextual defeasible logic and then prescribes treatment and severity level. After allotting severity level and treatment, the system sends this information back to the database. The hospital has complete access to this database and will take action accordingly.

**Figure 2:**
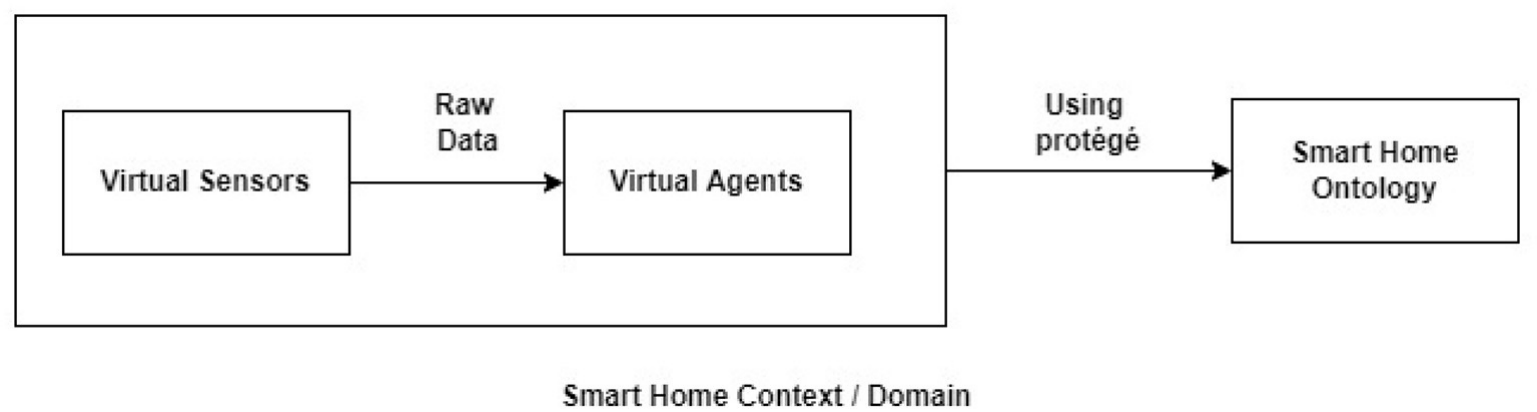
Smart home system.

These systems, which are a smart home system and smart hospital system, then send their received data to the smart decision-making system embedded in the smart hospital system so that the smart decision-making system could form a diagnosis of the patient's current health and the necessary treatment. Since multiple heterogeneous contexts/systems/domains are involved in this framework, structuring the overall system and data is crucial. Therefore, ontology is used for this purpose. To conclude, the smart decision-making system needs some reasoning mechanism. For this purpose, contextual defeasible reasoning is used. The flow/sharing of information between heterogeneous contexts is done and maintained using rules. Figure 2 explains the data acquisition and sharing in a smart home environment. The virtual sensors embedded in the smart home or worn by the patient inside the smart home collect raw data and send it to the virtual agents in a continuous manner. The virtual agents, which are intelligent, use their reasoning capabilities to generate an alert if a certain reading is obtained which is above or below a certain threshold. The agents alert the smart hospital domain's smart decision-making system.

**Figure 3:**
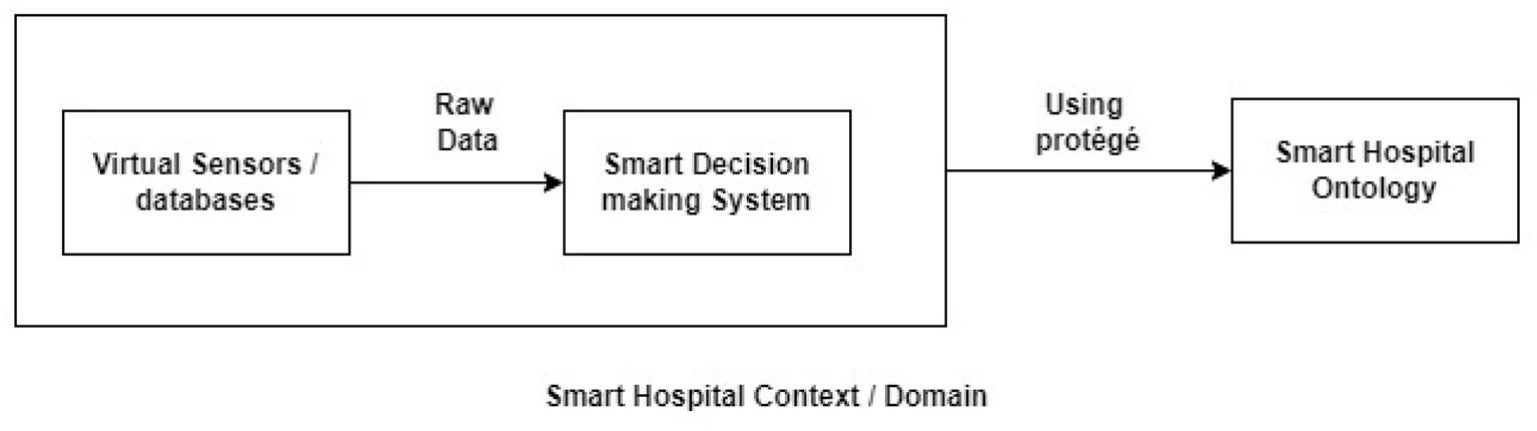
Smart hospital system.

Figure 3 explains the collection and transfer of data in a smart hospital environment. The virtual sensors embedded all over the hospital collect raw data and continuously share it with the virtual agents. The virtual agents use their reasoning capabilities and generate an alert if a particular acquired data reading is beyond or below a certain threshold. This alert generated is sent to the smart decision-making system by the agents implemented in the smart hospital domain. Databases containing information about the patient's allergies, and insurance, are also involved in this context or domain. They send the relevant data to the smart decision-making system whenever needed.

Intelligent virtual agents carry out the step that includes data acquisition of the MCS. The virtual agents take into account all data that real physical agents measure. The proposed system includes Multi-Context Systems consisting of several heterogeneous contexts. After this, in order to map the acquired data into the different domains of the system, ontologies are developed with their own set of rules. The developed ontologies are then mapped to their correlated description logic. Ontologies based on descriptive logic use the distributed description logic for interconnection *via* mapping rules. Once the system gets settled, the flow of information begins by heterogeneous contexts interactivity. Contextual defeasible reasoning (CDL) is applied to the system after the information flow and used to handle any inconsistencies.

**Figure 4:**
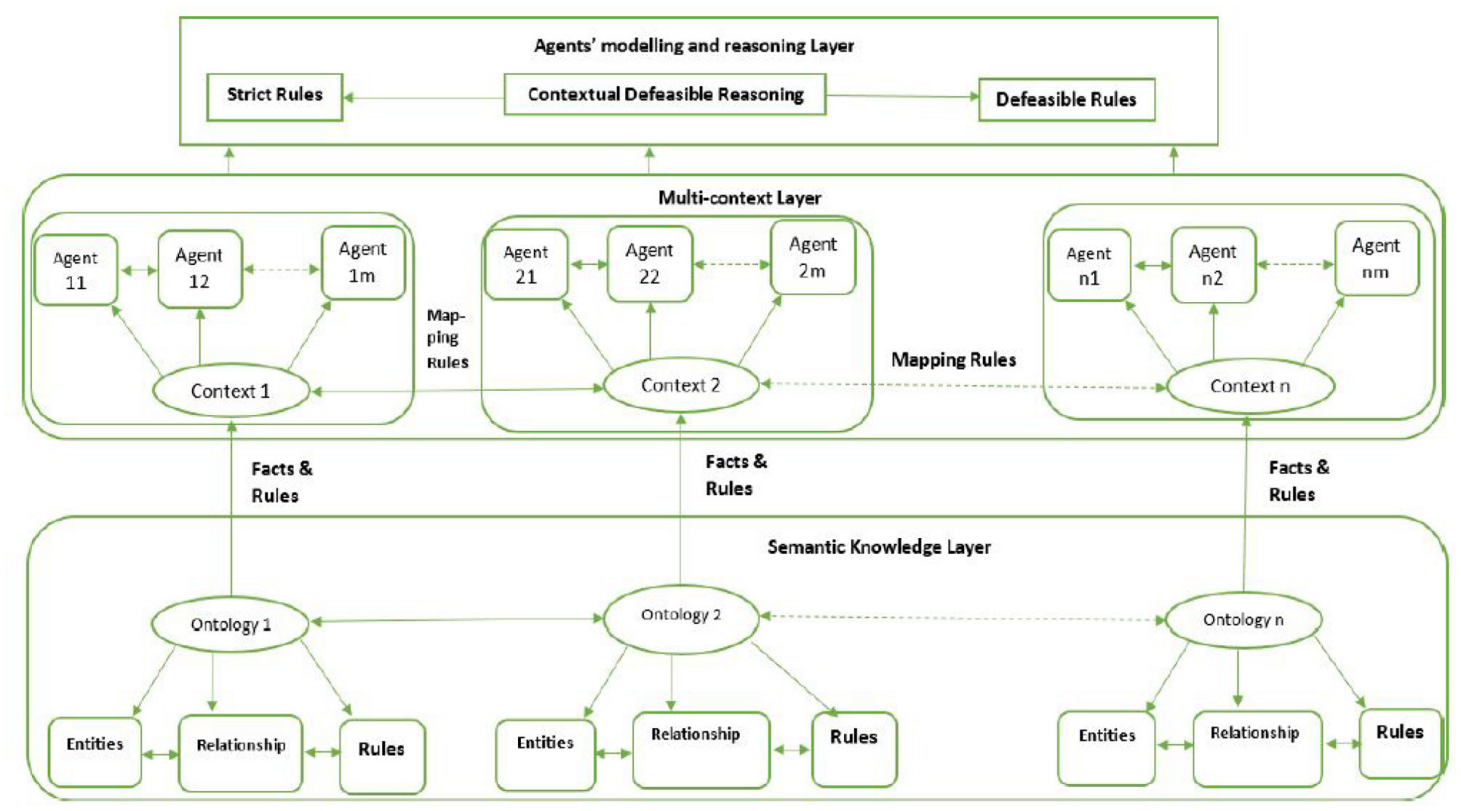
Proposed system layered architecture.

After this, contextual defeasible reasoning is applied to the system to handle any inconsistencies. CDL uses its facts, strict, defeasible, and defeater rules and preferences to solve these issues. The proposed framework verification is achieved by intending to develop a prototype or with the help of simulation. At the moment, implementing the proposed system is difficult due to financial and technical constraints. Our emphasis is mainly on research and partially on development. The development done is mainly for testing purposes. We are assuming that sensors are collecting the data. This data is modeled using agents. The reasoning performed on this system is done through contextual defeasible reasoning. Since our system is heterogeneous, therefore, initially, the agents are defined in different heterogeneous contexts. These heterogeneous contexts communicate with each other *via* mapping rules. Figure 4 explains the overall layered architecture of the proposed framework.

## 4. Framework Development

### 4.1. Context Development

To check the correctness of this work, we have developed a case study about a person who has Parkinson's disease (a neurodegenerative disease). The following two contexts have been developed for this case study.

#### 4.1.1. Context 1: Smart Home

**Figure 5:**
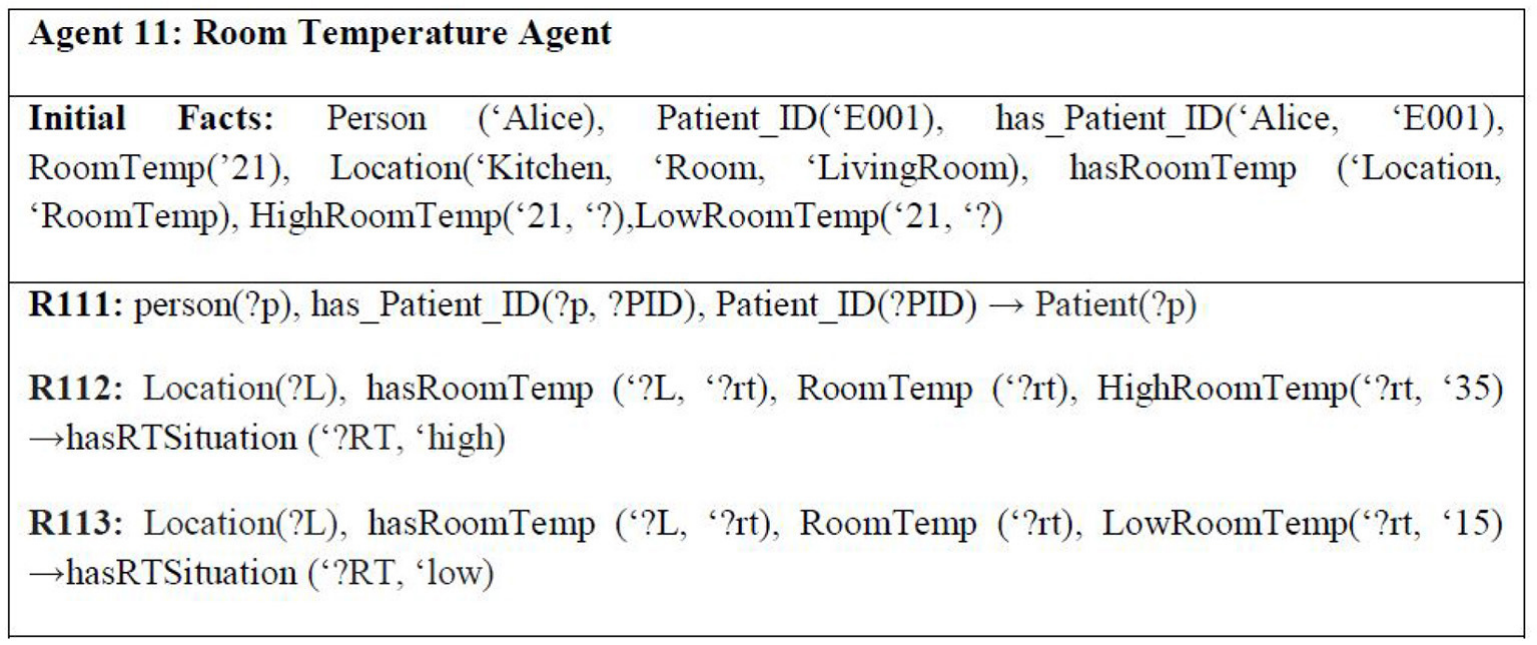
Example rules of context 1 smart home.

Since memory loss is the dilemma of Parkinson's disease, Alice's house has been embedded with various sensors. One of them is the motion detector sensor for sensing the current position of Alice, i.e., whether she is sitting, standing, has fallen over, or is showing symptoms of Slowed movement (bradykinesia) (21) one of the cardinal manifestations of Parkinson's disease, and for determining the current location of Alice in her house, i.e., Alice is in which room of her house is currently. This sensor is embedded in every room of her house. There are also smoke detection sensors to detect the presence of smoke or fire in the house. Another sensor being used is the carbon monoxide (CO) detection sensor that helps in alerting about emergencies, such as a person has turned on the gas of the stove but forgot to light it. Carbon monoxide is an extremely gas, and interaction with it for an extended period can be injurious to health (22). The smoke detectors are embedded everywhere in the house. However, the carbon monoxide detector is embedded in the kitchen only. On average, the carbon monoxide level recorded in houses with gas stoves is between 0.5 and 5 parts per million (ppm). However, in houses with gas stoves that are properly fixed, the normal recorded level is between 5 and 15 ppm, but the level may be 30 ppm or higher if the gas stoves are not correctly fixed (22). Figure 5 shows some example rules for the smart home context.

#### 4.1.2. Context 1: Smart Hospital

In addition to home embedded sensors, some sensors are worn by Alice. These sensors are the heart rate monitoring sensor to detect fluctuations in heart rate, body temperature sensors for measuring changes in body temperature, and surface electrodes sensors for measuring electrocardiography (ECG), electromyography (EMG), or electroencephalography (EEG). They are used to monitor the electrical activities of the heart, muscles, and brain, respectively, directly from the skin's surface, eventually providing information about rigidity in the skin and detecting body tremors, both of which are prevalent symptoms of Parkinson's disease.

**Figure 6:**
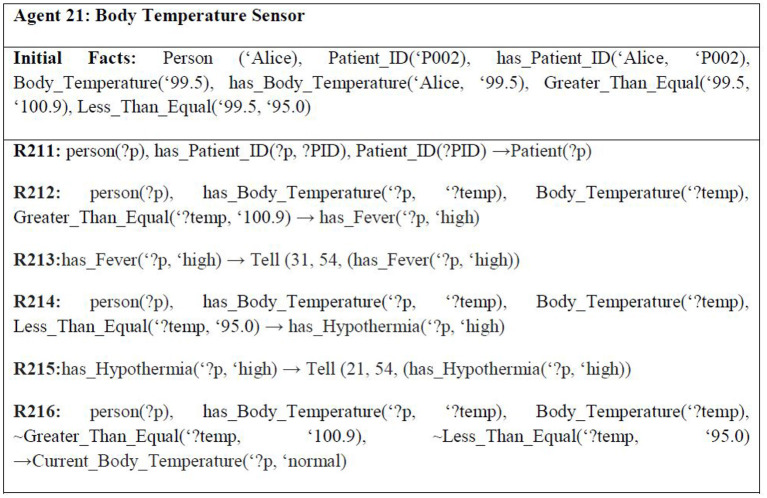
Example rules of context 2 smart hospital.

It must be mentioned here that a specific range of “normal” values is associated with each of these sensors. In case a reading value of a sensor goes above or below this range, an alert is issued by the sensor. The normal range for a heart monitoring sensor is 60–100 bpm (precisely 82 bpm). Usually, an adult has a body temperature between 97 and 99 F (22). For ECG sensors, normal electrocardiography intervals are 0.6–1.2 s. Based on the muscle, the normal EMG range is between 50 μV and 30 mV. An average adult has EEG readings between 8 Hz and higher. For EMG, a reading of 7 Hz or less is considered abnormal in awake adults. However, they are considered normal in children, or sleeping adults (23). Figure 6 shows some example rules for the wearable device context.

### 4.2. Agent Development

Nine intelligent virtual agents are designed for the proposed framework. Their description is provided below.

- Systolic Blood Pressure (BP) Agent: For monitoring the systolic BP
- Diastolic BP Agent: For monitoring the diastolic BP
- Body Temperature Agent: For monitoring the body temperature
- Heart-Rate-Agent: For monitoring the Heart Rate
- EEG-Agent: For monitoring the EEG
- ECG-Agent: For monitoring the ECG
- EMG-Agent: For monitoring the EMG
- Carbon Monoxide-Agent: This agent monitors the carbon monoxide level
- Carbon Dioxide-Agent: This agent monitors the carbon dioxide level
- Room-Temperature-Agent: This agent monitors the room temperature

### 4.3. Ontology Development

**Figure 7:**
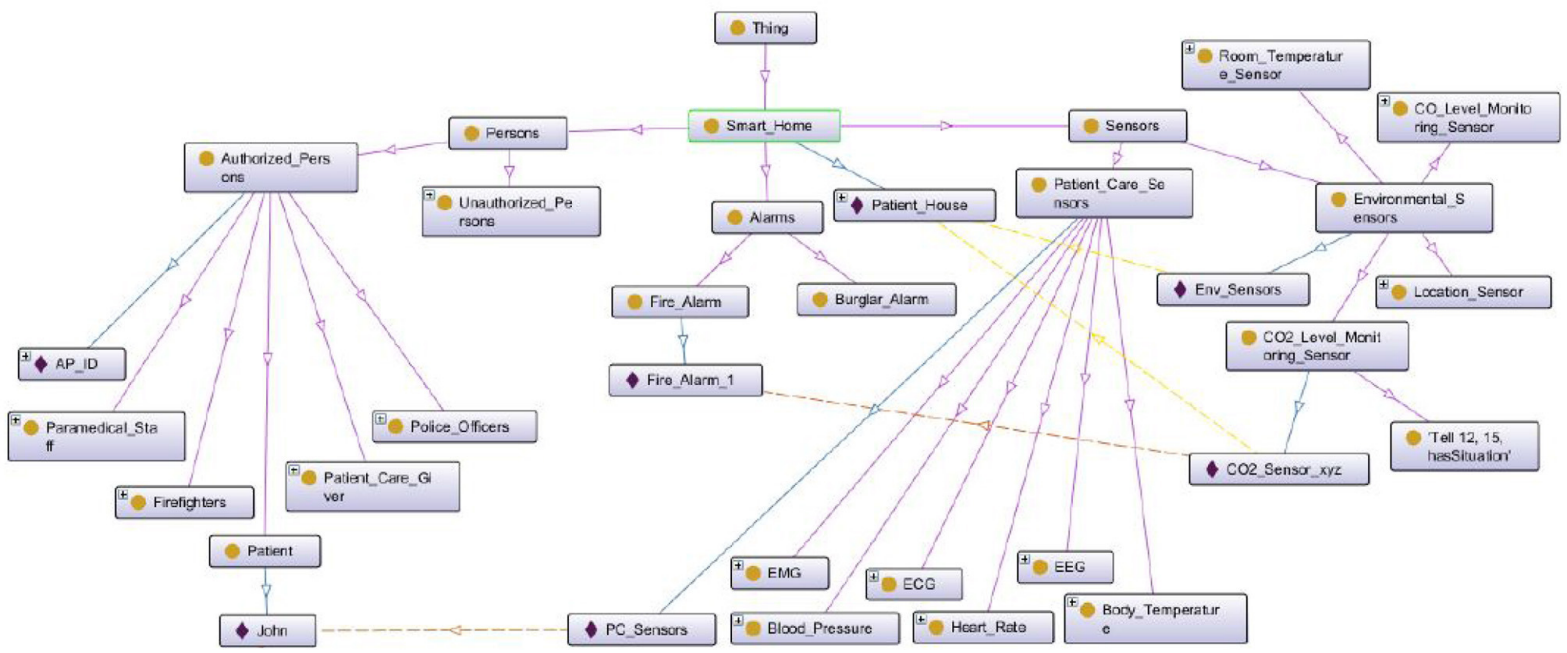
Smart home ontology.

For system modeling, contextual information is obtained from multiple ontologies.To model distributed domains for the proposed system, a smart home ontology and a smart hospital ontology are developed for modeling the proposed framework. Fragment of the smart home ontology is shown in Figure 7.

**Figure 8:**
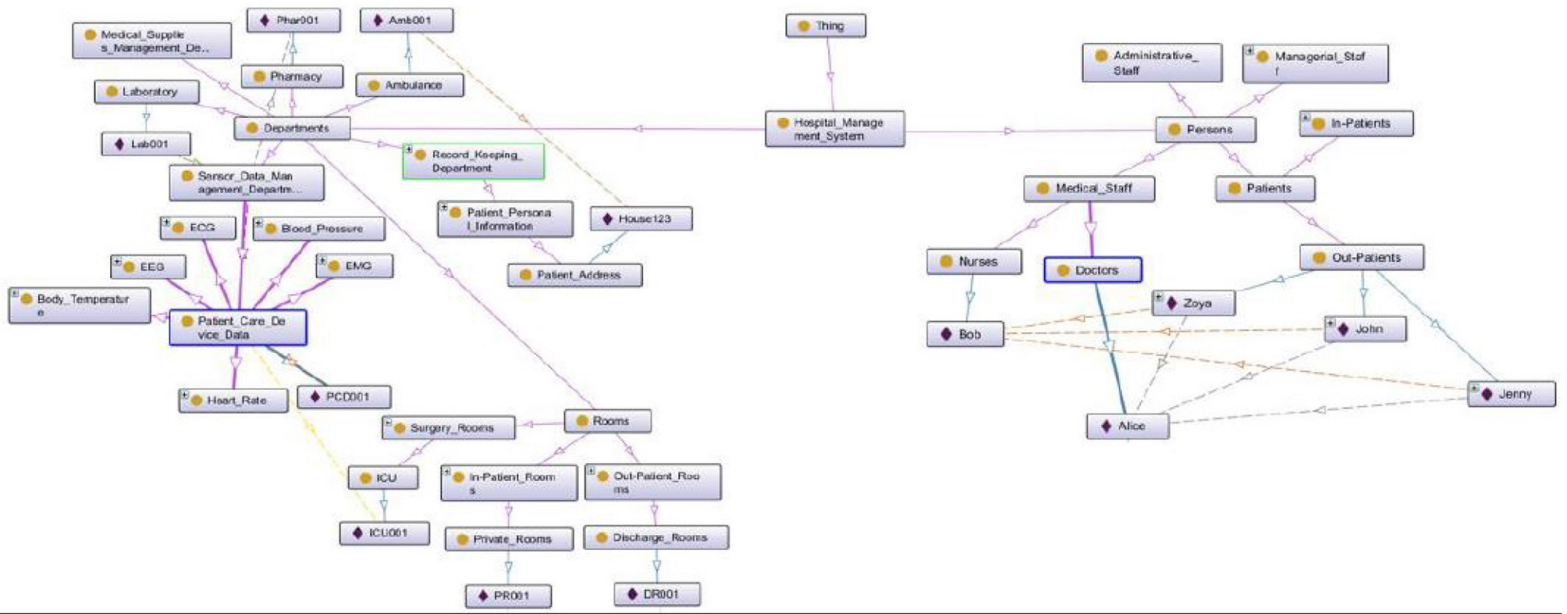
Smart hospital ontology.

The smart home ontology describes the flow of information in this context. The second ontology developed is the smart hospital ontology. This ontology describes the structuring of the smart hospital context and expresses the relations between different smart hospital context entities. It also describes the flow of information in this context. Fragment of the smart hospital ontology is shown in Figure 8.

In the proposed framework, agents acquire contextual data either from a single ontology or multiple ontologies based on the system's design. Mapping rules have been designed to model the flow of information between the contexts involved in the framework. To manage inconsistencies in such systems, priorities are assigned to the mapping rules, with the rule having the higher priority being fired. Class hierarchies of the developed two ontologies are also shown in Figure 7.

## 5. Temporal Logic Formalism

There are two types of models in temporal logic. Linear temporal logic (LTL) and computational tree temporal logic (CTL*) (24). In our work, we use CLT* instead of LTL because LTL is based on a single computational path (25).

The semantic of TL is defined by CLT^*^ (26). The changing of the current state, i.e., state to -> state from and state from -> state to, is represented by the ω-tree shape (27). The states correspond to the agent knowledge base and communication mechanism. The agent fires multiple rules in a multi-agent system to reach the required state that is acceptable as an outcome. Each agent may drive new context whenever it matches the pre-defined rules. When the agent acts on its actions, the system moves to the next state. If the agent did not acquire the data, the agent tells its corresponding agent about the context, and then the system moves to the other state.

### 5.1. Current Situation Observation

When an agent gets something from its corresponding sensor, it checks it to map the acquired context with the internal knowledge.

#### 5.1.1. Pre-defined Rules

- G Ag^*i*^ (ϕ, t1) where ϕ ∈ IK^*i*^ - (i)
- G Ag^*i*^ (¬ϕ, t1) where ϕ ∈ IK^*i*^ - (ii)

In the rule mentioned above i and ii, globally, agent i gets information from the environment which belongs to the agent's internal knowledge IK or information has not been detected by agent i but belongs to internal knowledge. Here, IK^*i*^ represents internal knowledge. For instance, when the fire information is acquired to the agent thus the fire context belongs to the agent's internal knowledge; else, not fire belongs to it.

- F [∋ Tell (i, j, ϕ)] - (iii)

In rule iii, it states in the future, on some existing states s' agent^*i*^ believes in telling agent^*i*^ about some acquired context information, so the agent^*i*^ can be auto-trigger.

- Ag^*i*^ (ϕ, t1) where ϕ ∈ HI - (iv)

Agent^*i*^ acquired the state formula concerning t1, and it is true, and that state formula belongs to acquire HI. For example, after selecting the optimal plan, agent^*i*^ has the information about calling the humanitarian assistance for human involvement to give the alert related to the unauthorized entity, as shown in rule iv.

#### 5.1.2. Successful Events

- ϕ i ε ψ i - (v)

When agent^*i*^ acquires the context ϕ about one state formula or agent^*i*^ acquires the context ψ about other state formula or both. In every way, it would be a successful case. For example, if agent^*i*^ gets the information about an animal who is on-site where the camp of soldiers are, or agent^*i*^ gets the information about detecting the unauthorized entity or both. It would be a successful case, as shown in rule v.

- Agi (¬ϕ, t1) where ϕ ϵ IK^*i*^ -(vi)

Agent i has not detected any information, and it does belong to internal knowledge; therefore, it is the case of partial success, e.g., an agent did not get the information about detecting any unauthorized entity, is still a partial success case, as shown in rule vi.

- Ask (j, i, ϕ) ε Tell (i, j, ϕ) - (vii)

In rule vii, agent^*i*^ can ask agent^*i*^ about the specific context, or agent^*i*^ has the pre-defined rule to tell the specific context to agent^*i*^. For example, agent^*i*^ has the rule to ask agent^*i*^ about detection, or agent^*i*^ has the rule to tell agent^*i*^ about the detected entity.

#### 5.1.3. Current Situation Step of Actions

After the agent checks the acquired information is true or not, it has the step of actions to follow to act efficiently.

- Xi (α, ti) - (viii)

In rule viii, after matching the predefined rules, the very next thing is, agent^*i*^ has to perform θ certain actions α. For instance, if the unauthorized entity context detection is true, then agent^*i*^ has actions α to perform θ in a specific time t_*i*_.

- F [∋ i (α (HI), t_*i*_)] - (ix)

After matching the pre-defined rules, in the future, in some existing states, an agent^*i*^ has the actions to perform HI requirements. After selecting the optimal plan, agent^*i*^ would contact the HI to prevent the situation from any damage, as shown in rule xi.

#### 5.1.4. Task Priorities

- DL (H) iff ϕ i ∪ ψ j - (x)
- DL (S) iff ϕ i ∧ ψ j - (xi)

The danger level DL is always high (H). If the one-state formula is detected ϕ unless the other state formula ε is detected, DL changes to severe (S). For example, unauthorized entity ϕ with any weapon at the site ψ is severe DL, as shown in rules x and xi.

## 6. Simulation of Proposed System

This section explains the case study, we have developed for the validation of our system. For its implementation, we have used the Netlogo simulation tool. Netlogo is used for modeling, and it teaches the concept of agents like turtles, patches, observers and links, etc., it is used in different kinds of scenarios like in gaming, or if people built a model for a war to check it whether it is correct or not, or/ and in disaster management scenarios, etc. It is open-source software that includes an interface, commands to create an agent model and execute them. It has built-in commands and reports that they call primitive, and the command reports created by a programmer called procedure (28, 29). To achieve the perfect communication between agents, FIPA protocol was used (30).

For example, agent 1 tells agent two about an “Emergency Situation” by sending “Abnormal BP detected” in the message.

["Emergency Situation" "sender:1""content:" "Abnormal BP Detected" "receiver:2."]

**Figure 9:**
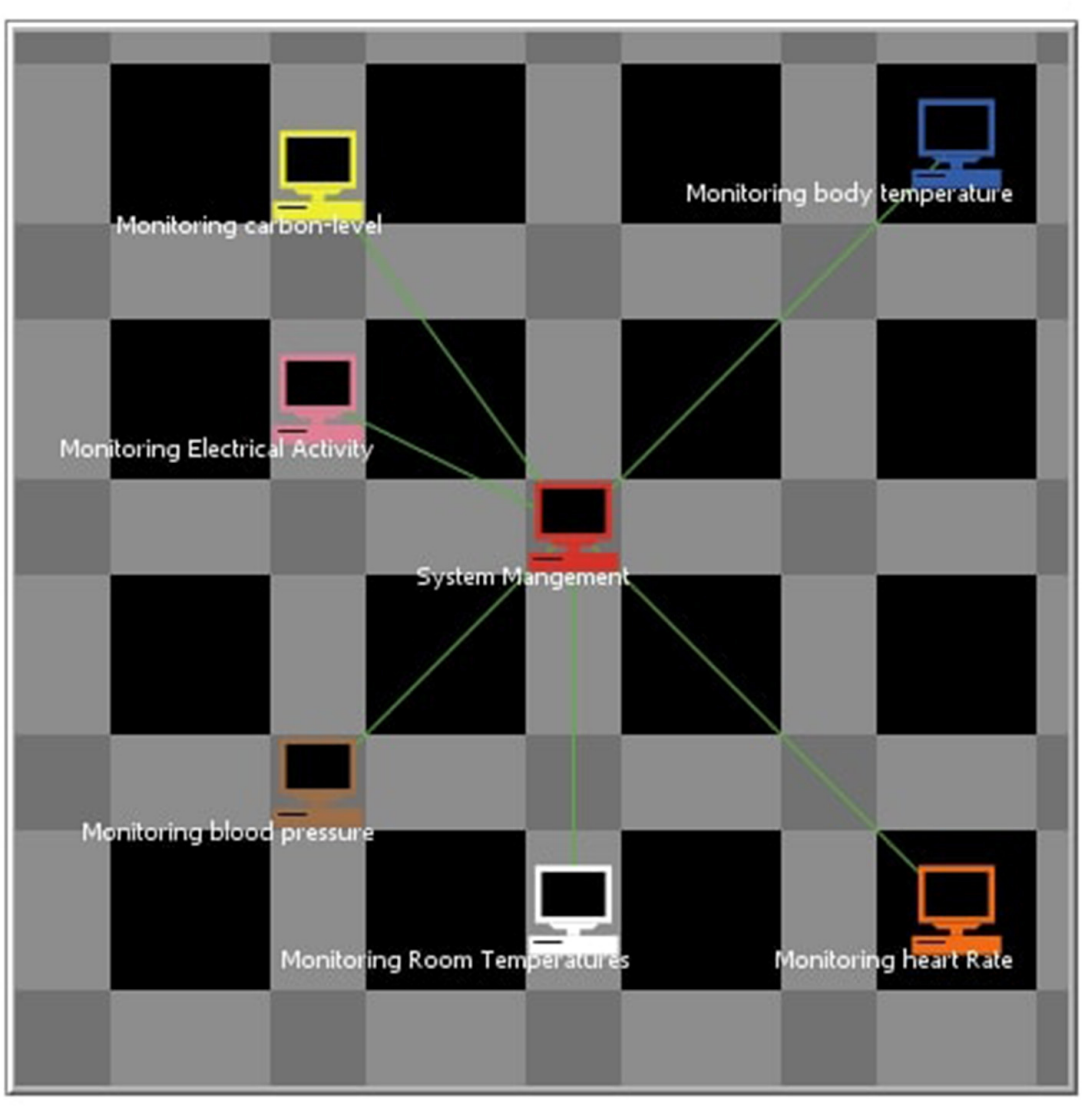
System interface.

Figure 9 shows the interface of our system. We created seven context-aware agents named controller agent, BP agent, body temperature agent, heart-rate agent, electrical-activity agent, carbon-level agent, room-temperature agent. Each of them has their unique functionality based on their own set of facts and rules, also perform reasoning and take decisions intelligently. These agents can communicate in NetLogo as they are connected by creating a link.

**Figure 10:**
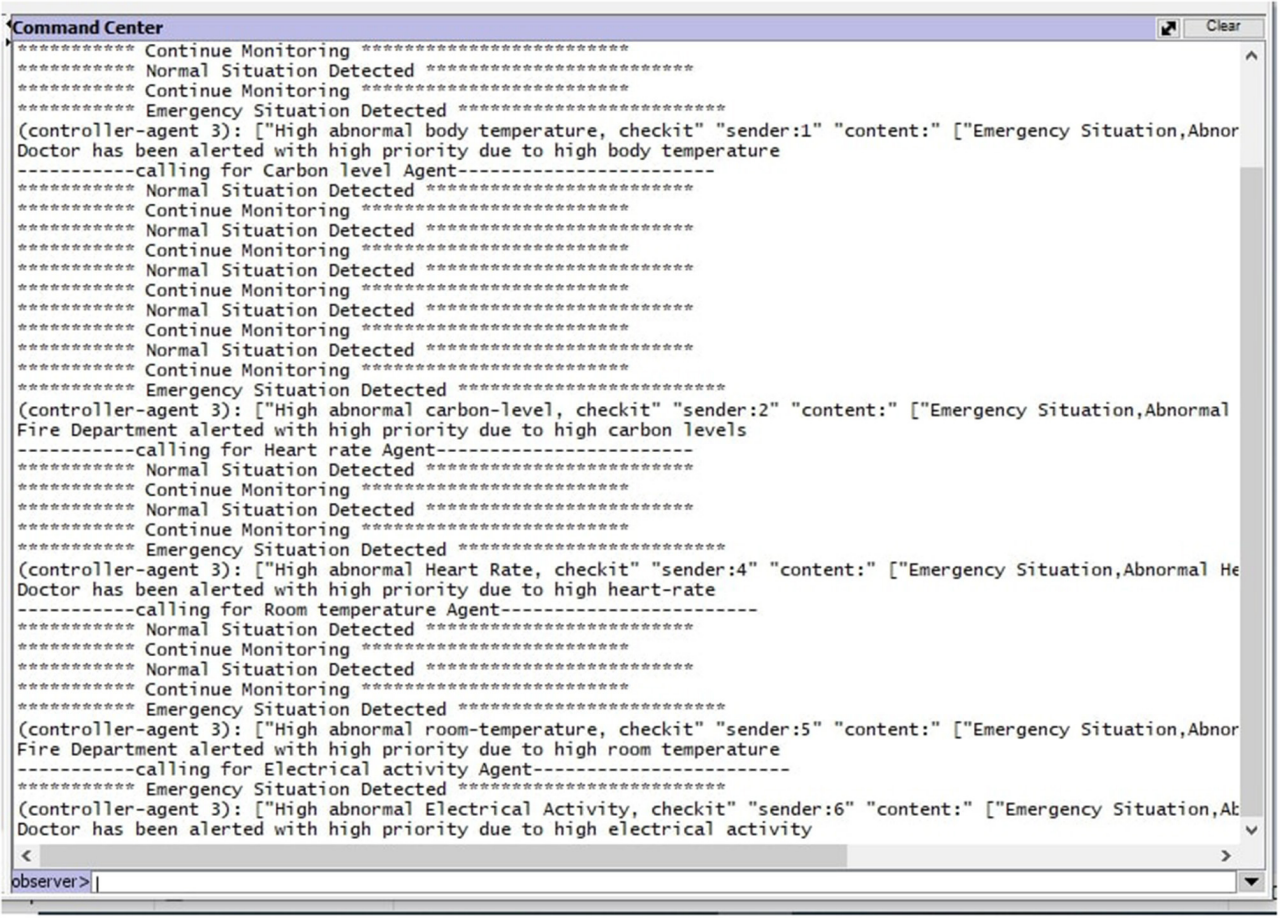
System interface.

Figure 10 shows the execution of the system. During the execution, the system continuously monitors the patient's situation as “Continuously Monitoring.” When any “Emergency Situation” is detected controller agent gets to inform a respective agent that is highly abnormal body temperature detected. After that controller agent sends a message to the doctor by alerting the situation “Doctor has been alerted with high priority due to high body temperature.”

## 7. Algorithm

The algorithm has two parts. The first part (Algorithm 1) is invoked as soon as the system begins. However, invoking the second part of the algorithm (Algorithm 2) depends on obtaining an abnormal value (above or below the pre-defined normal range). In Algorithm 1, the agents collect contextual information from their environment. Until that contextual information remains within a specific range (the value obtained is above the lower pre-defined range and below the upper pre-defined range), the system generates no alert and usually works. However, if any agent obtains a value that does not fall within the pre-defined normal range (the value obtained is either below the lower or above the upper pre-defined range), the system generates an alert (Algorithm 2) invoked.

**Algorithm 1:**
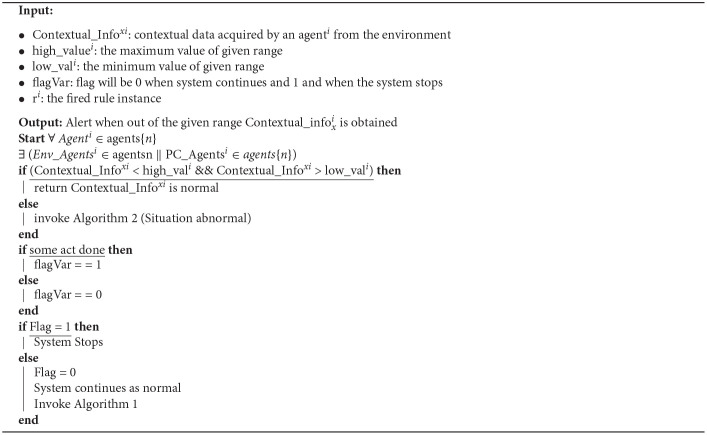
Obtaining of Data.

**Algorithm 2:**
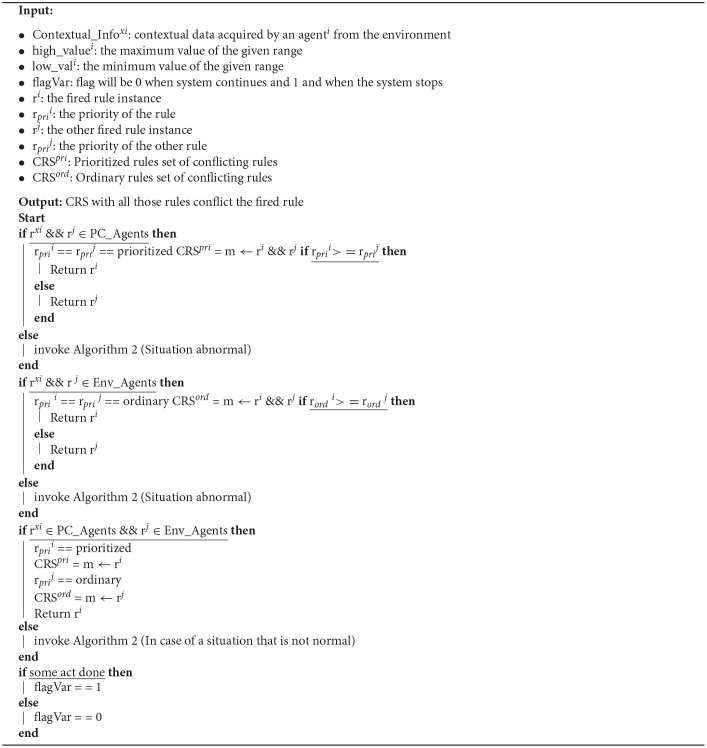
Conflicting Rule Set Creation

In Algorithm 2, the priority of the rule instances is fired, and the rule with the highest priority is chosen. The following action, alerting the corresponding authority, depends on the rule instance fired. In the case of PC_Agents rule instances, the doctors, caretakers, and the next of kin are alerted. On the other hand, when the rule instances fired are from Env_Agents, then the fire department, emergency response unit, and the police department are alerted.

This alert is sent to the corresponding authority by the controller agent of the system. The controller agent is the primary agent of the system controlling all information flow. All the contextual information obtained by any agent is sent directly to the controller agent. In case of an emergency (an abnormal value), the agent sends an alert to the controller agent, and the controller agent then contacts the corresponding authority. The contact can be made in any number of ways, and it can be a text message, an email, a notification, a phone call, or a WhatsApp message. The mode of communication is chosen by the corresponding authority and then included as a command in the memory of the controller agent.

Once an alert has been generated, it is up to the corresponding authority to either take action or not do anything. The corresponding authority solely takes this decision. The reason for this decision goes beyond the scope of this work and the author's domain. If the corresponding authority believes action must be taken, it alerts the system, and the flag's value is set to 1. The flag is a variable that can have only two values, either 1 or 0. each value corresponds to a separate course of actions. If the flag's value becomes 1, the system knows that the corresponding authorities have taken over. In such a case, the system will halt and wait to be rebooted by the corresponding authority. However, if the corresponding authority believes that no action needs to be taken, it does not alert the system, and the flag's value is set to 0. If the flag's value becomes 0, then the system knows that the corresponding authorities have not taken over now, and they will continue to work as usual (continue gathering contextual information). In such a case, the system will not halt. The working of the algorithm in case a single rule is fired or in case multiple rules are fired simultaneously is described below. It must be mentioned here that by default, the priority of patient care agents (PC_Agents) is more than environmental agents (Env_Agents).

### 7.1. Case 1: Single Rule Instance Fired

When a single rule is fired, the system will function according to that rule without any conflict or issue.

### 7.2. Case 2: Multiple Rules Instances

When multiple rules are fired, then the set CRS_*pri*_ will be developed. All the conflicting rules will be included in the CRS_*pri*_ set, and ultimately the rule with the highest priority will be selected.

## 8. Limitations and Future Scope

Since the inconsistencies in heterogeneous knowledge sources systems usually do not have a single reason for occurrence but arrive from the interaction of heterogeneous knowledge bases, therefore, conflicting, or contradictory information is a major reason for an inconsistent system. According to the best of our knowledge, the existing approaches propose no hard and fast method for assessing inconsistencies in MCS as so far, no proposed method is flexible enough to accommodate the criteria of various applications. A new approach is needed to provide a single platform to overcome the issues of inconsistency due to conflicting or contradictory information. This new approach must have a reasoning mechanism to handle the issues while having a solid logical base to perform sound reasoning. Since these issues occur due to knowledge sharing among heterogeneous contexts, a mechanism is needed to represent the knowledge formally. There can be multiple heterogeneous contexts in a multi-context system. Thus having a mechanism for structuring such a system in an organized manner is extremely necessary. The proposed framework fulfills all the requirements mentioned above by using ontology to structure the system, description logic for knowledge representation, and contextual defeasible reasoning for performing reasoning. This work is beneficial for patients who require continuous monitoring of their health, but they cannot afford to stay in a monitoring facility or do not have enough resources to accommodate all their patients. In such cases, this system can be helpful as it will allow the patient to be under supervision continuously by the agents, and in case of an emergency, the authorities will be instantly notified. Another implementation of this system is for those patients who may not need continuous monitoring but are suffering from terminal illnesses such as Parkinson's disease. In these cases, the disease can act up at any moment. The exact time of such situations cannot be predicted. In these scenarios, the patients can be monitored in the safety of their homes, and in case of emergencies, the medical authorities will be notified immediately. In other domains, this system can also be implemented. In disaster management systems, this system can be used to manage disasters such as fire eruptions, carbon dioxide or carbon monoxide leakage, the occurrence of flood, etc.

## 9. Conclusion

In this work, an ontology-driven formalism has been proposed for handling inconsistency in a highly dynamic environment. The proposed framework and application differ from other approaches because it uses contextual defeasible logic to solve inconsistencies. The proposed framework can provide a single platform to overcome the issues of inconsistency occurring due to contradictory information in a multi-context system. Contextual defeasible reasoning has been chosen to perform reasoning on the system as it can handle inconsistency issues by providing a solid logical base to perform sound reasoning. Since the issue occurs due to sharing knowledge among heterogeneous contexts, description logic (DL) and distributed description logic (DDL) mechanisms represent the knowledge being shared formally. While an ontology-based modeling approach has been adopted to model the multiple heterogeneous contexts in a multi-context system. Thus, the proposed framework provides a formalism of multi-agents based on that can handle conflicting information in an environment that is highly decentralized in nature.

## Data Availability Statement

The original contributions presented in the study are included in the article/supplementary material, further inquiries can be directed to the corresponding author/s.

## Author Contributions

AJ and SA: conceptualization. SA: data curation. AJ: formal analysis, investigation, methodology, and software. SB and MN: funding acquisition. KS and MN: project administration. RA and MN: resources and writing—review and editing. AM, AJ, and KS: supervision. SA, AM, and RA: validation. SA and KS: visualization. All authors contributed to the article and approved the submitted version.

## Funding

This paper was supported by TU-Dresden, Dresden, Germany.

## Conflict of Interest

The authors declare that the research was conducted in the absence of any commercial or financial relationships that could be construed as a potential conflict of interest. The handling editor declared a past co-authorship with one of the author AJ.

## Publisher's Note

All claims expressed in this article are solely those of the authors and do not necessarily represent those of their affiliated organizations, or those of the publisher, the editors and the reviewers. Any product that may be evaluated in this article, or claim that may be made by its manufacturer, is not guaranteed or endorsed by the publisher.
